# Do changes in working hours increase stress in Japanese white-collar workers?

**DOI:** 10.3389/fpubh.2023.1076024

**Published:** 2023-02-01

**Authors:** Masaki Ozawa, Tatsuhiko Anzai, Takashi Yamauchi, Kunihiko Takahashi

**Affiliations:** ^1^School of Medicine, Tokyo Medical and Dental University, Tokyo, Japan; ^2^Department of Biostatistics, M&D Data Science Center, Tokyo Medical and Dental University, Tokyo, Japan; ^3^Department of Public Health and Environmental Medicine, The Jikei University School of Medicine, Tokyo, Japan

**Keywords:** stress, working hours, white-collar, longitudinal study, full-time workers

## Abstract

**Introduction:**

High stress at work is associated with negative health outcomes for workers, making stress prevention a critical challenge. Overtime work is an influential stress factor. This study, therefore, aimed to longitudinally evaluate how stress increased depending on changes in working hours among Japanese white-collar workers.

**Methods:**

We targeted 3,874 participants who were full-time workers and were recognized as having low stress in a web-based cohort in 2018 (T1) and 2019 (T2). We performed univariate and multivariate logistic regression with the following variables: years of experience, years of education, medical background, income, and roommates.

**Results:**

We observed a greater increase in stress among female who worked 41–50 h per week at T1 and more than 50 hours per week at T2, and those who worked more than 50 h per week at T1 and 35–40/41–50 h per week at T2, compared to those who worked 41–50 h per week both at T1 and T2, with odds ratios (ORs) and 95% confidence intervals (95% CI) of OR = 2.09, 95% CI (1.18, 3,70); OR =1.86, 95% CI (1.14, 3.03), respectively. However, no association between change in working hours and stress was found among male.

**Discussion:**

These results show that reducing stress requires decreasing working hours as well as identifying factors that lead to high stress.

## 1. Introduction

High stress is generally viewed as harmful to an individual's physical and mental state (1). While there are several types of stressors, recent studies have shown that high stress at work is associated with increased risk of negative health outcomes, including impaired cognitive function, depression, cardiovascular diseases, and poor sleep quality (2–4). Hence, the prevention of work-related stress is widely recognized as a critical challenge for occupational health and safety (5).

Overtime work is generally thought to be one of the most influential risk factors for high stress (6). Typically defined as 55 working hours or more per week, overtime work is also a risk factor for health-related problems such as coronary heart disease (7). High job demands can result in burnout and other risks as a consequence of overwork (8). It is believed that stress increase as a result of these factors. Overtime work of more than 40 h per week was associated with higher levels of stress in a representative sample of the United States population (9). In a study analyzing the impact of long working hours on psychosocial stress response among white-collar workers in one Korean company, working 60 or more hours per week was significantly associated with higher stress compared to working 40–44 h per week (10). Among 59,021 Japanese workers in 117 companies, the length of working hours was positively associated with various higher stress responses, including “irritability,” “fatigue,” “anxiety,” “depression,” and “somatic responses” for both genders (11). In fact, in Japan, where overtime work has been prevalent, the government has considered stress management to be of paramount importance. Japan is the first country in the world to make annual stress check tests legally mandatory in a workplace with 50 or more employees, since 2014 (12, 13). The stress check is performed using the Brief Job Stress Questionnaire (BJSQ), developed based on the demand-control-support model (14, 15).

In addition, the Japanese government is trying to reduce long working hours as one measure to address the shortage of workers (16). The outbreak of the novel coronavirus infection may have increased the working hours and various other burdens on some workers. Even previously stress-free individuals may have been exposed to new stress risks. However, previous research has shown that only those currently working long hours tend to have high stress. It remains to be seen which people will likely become highly stressed in the future, depending on changes in working style. Understanding who is prone to high stress among those who are not under high stress may help employers implement measures to prevent high levels of stress.

Therefore, we longitudinally examined how many participants were identified as having high stress after 1 year based on a survey conducted at two time points among a representative sample of Japanese workers. To focus on the increase in stress between the two points, we targeted those identified as having low stress at the first point. Additionally, we examined differences in how stress increased depending on changes in working hours by gender, considering that women and men in Japan have significantly different ways of working (17). As in the previous study mentioned earlier, we targeted white-collar workers aged 20–64 years, which is also validated by the fact that the most common type of work in Japan is white-collar.

## 2. Materials and methods

### 2.1. Data and materials

We used the same data as in a previous study (18). A web-based cohort survey regarding the occupational safety of 30,000 workers was conducted twice in October 2018 (T1) and October 2019 (T2) in collaboration with a research company in Tokyo, Japan. This company has one of the largest online research panels in Japan with over 1.8 million voluntarily registered panelists. To minimize selection bias, we selected a sample of Japanese workers aged 20–64 years based on the composition ratio of workers by industry, gender, and age in the Labor Force Survey by the Japanese Ministry of Internal Affairs and Communications. The research company randomly sent an e-mail invitation to registered workers to participate in the study. Workers who provided web-based informed consent were selected to participate in the survey. They completed a self-reported online questionnaire consisting of questions regarding demographic, job-related, and life-related variables, as well as health-related outcomes. The study protocol was approved by the Institutional Review Board of the Jikei University School of Medicine, Tokyo, Japan, in 2018 [No. 30-153(9174)].

### 2.2. Measures of stress and working hours

We selected full-time employees (over 35 h per week as the average working hours during the last 6 months) who reported being professional or general clerks, based on the Japan Standard Industrial Classification established by the Japanese Ministry of Internal Affairs and Communications.

We targeted those employees identified as having low stress at T1 using the BJSQ, a tool widely used to evaluate job-related stress in Japan (19). In this study, low-stress population was identified as the population not determined to have high stress according to the following criteria. The BJSQ comprises 57 items that assess (A) job stressors, (B) psychological and physical stress responses, and (C) buffering factors. (A) Job stressors include quantitative job overload, qualitative job overload, physical demands, job control, skill utilization, interpersonal conflict, poor physical environment, suitable jobs, and intrinsic rewards (17 items). (B) Psychological and physical stress responses include lassitude, irritation, fatigue, anxiety, depression, and physical stress responses (29 items). (C) Buffering factors include supervisor support, co-worker support, and support from family and friends (9 items). Those whose scores in section (B) were over 76, or whose total scores in sections (A) and (C) were over 75 and scores in section (B) were over 62 were defined as having high stress (20).

The outcome measure of the present study was to check whether each participant was defined as having high stress at T2 in the following two ways: when the score criterion in section (B) only, representing physical reaction, was met (criterion A); when the score criterion in sections (A) and (C) were met even if the score criterion in section (B) was not met (criterion B).

We grouped self-reported average working hours per week in the last 6 months into seven categories based on how the hours changed between T1 and T2: 35–40 h/week in T1 remained the same or increased in T2, 41–50 h/week in T1 decreased, remained the same, or increased in T2, and 51 h/week or more in T1 decreased or remained the same in T2. Since many Japanese workers are required to work the full 40 legal working hours, we considered working 40 h plus a few hours of overtime per week as the most standard way of working. Therefore, we set the reference group as those who worked 41–50 h per week in both T1 and T2.

### 2.3. Other variables

We categorized years of experience in the job into four categories: 5 years or less, 6–10 years, 11–20 years, 21 years or more. We split years of education into two categories based on whether each participant graduated from college. We assessed medical background according to whether each participant was using medications. We divided income into four categories: those whose income at T2 increased, decreased, or remained the same compared to T1 and those who did not want to answer. We grouped roommate status into two categories: those whose number of roommates remained the same or increased/decreased.

### 2.4. Statistical analysis

First, we summarized the demographic and baseline characteristics of those who met the eligibility criteria for each category of working hours. Second, we conducted univariate logistic regression to determine whether participants had high or low stress at the second point based on criteria A and B, as well as only criterion A as the objective variable, and how working hours changed between the first and second points according to the classification mentioned above as the explanatory variable. We also conducted multivariate logistic regression with years of experience, years of education, medical background, income, and roommates as other variables. In these model analyses, crude/adjusted odds ratios (ORs) with 95% confidence intervals (CIs) and *p*-values were calculated. The robustness of the results was confirmed by a sensitivity analysis in which the cut-off for working hours was changed from 50 to 60 h. Statistical significance was set at *P* < 0.05. All analyses were conducted using STATA version 17.0 (STATA Corp., College Station, TX, USA).

## 3. Results

Of 7,012 voluntary registered panelists who answered that they were general clerks or professionals, 5,165 worked an average of over 35 h per week during the last 6 months. Among them, 4,051 were identified as having low stress at T1. After excluding those who did not answer at either T1 or T2 or changed occupations between T1 and T2, data from 3,874 participants (2,167 female and 1,707 male) were analyzed.

The characteristics of the study participants, according to the category of working hours, are shown in Table 1. The mean age was approximately 43 years for both genders. About 45% of female participants and 72.5% of male participants reported having graduated from college; 27.7% of female participants and 58.4% of male participants reported being professional. Among female and male participants, the proportion of those identified as highly stressed based on criteria A and B was 14.6 and 14.3%, respectively. When identifying the highly stressed state using only criterion A, the rates were 13.1 and 12.7% for female and male participants, respectively.

**Table 1:** Summary of backgrounds and characteristics of female workers.

**Working hours** **(Hours per week)**	**T1 (October 2018)**	**35~40**		**41~50**			**51~**		**Total (female)**
	**T2 (October 2019)**	**35**~**40**	**41**~**50/51**~	**35**~**40**	**41**~**50**	**51**~	**35**~**40/41**~**50**	**51**~	
n		842	225	218	509	92	150	131	2,167
Age, mean (standard deviation)		44.1 (10.1)	42.6 (9.6)	42.6 (9.7)	43.5 (9.5)	42.3 (8.7)	41.4 (9.7)	42.2 (9.6)	43.3 (9.8)
Education year ≥16 years, *n* (%)		355 (42.2)	97 (43.1)	89 (40.8)	238 (46.8)	48 (52.2)	73 (48.7)	75 (57.3)	975 (45.0)
Professional, *n* (%)		157 (18.7)	75 (33.3)	72 (33.0)	154 (30.3)	30 (32.6)	55 (36.7)	57 (43.5)	600 (27.7)
**Years of experience**, ***n*** **(%)**									
≤ 5 years		256 (30.4)	71 (31.6)	77 (35.3)	148 (29.1)	33 (35.9)	47 (31.3)	38 (29.0)	670 (30.9)
6~10 years		199 (23.6)	54 (24.0)	36 (16.5)	103 (20.2)	27 (29.4)	41 (27.3)	33 (25.2)	493 (22.8)
11~20 years		239 (28.4)	67 (29.8)	66 (30.3)	158 (31.0)	22 (23.9)	40 (26.7)	32 (24.4)	624 (28.8)
≥21 years		148 (17.6)	33 (14.7)	39 (17.9)	100 (19.7)	10 (10.9)	22 (14.7)	28 (21.4)	380 (17.5)
**Income change**, ***n*** **(%)**									
No change		384 (45.6)	108 (48.0)	101 (46.3)	256 (50.3)	47 (51.1)	55 (36.7)	70 (53.4)	1,021 (47.1)
Increased		161 (19.1)	39 (17.3)	35 (16.1)	97 (19.1)	20 (21.7)	28 (18.7)	21 (16.0)	401 (18.5)
Decreased		125 (14.9)	29 (12.9)	33 (15.1)	64 (12.6)	12 (13.0)	25 (16.7)	19 (14.5)	307 (14.2)
No answer		172 (20.4)	49 (21.8)	49 (22.5)	92 (18.1)	13 (14.1)	42 (28.0)	21 (16.0)	438 (20.2)
The number of roommates changed, *n* (%)		46 (5.5)	15 (6.7)	16 (7.3)	33 (6.5)	3 (3.3)	15 (10.0)	12 (9.2)	140 (6.5)
With treatment, *n* (%)		59 (7.0)	14 (6.2)	9 (4.1)	34 (6.7)	5 (5.4)	13 (8.7)	18 (13.7)	172 (7.0)
High-stressed at T2 (all the region), *n* (%)		121 (14.4)	36 (16.0)	28 (12.8)	57 (11.2)	20 (21.7)	31 (20.7)	24 (18.3)	317 (14.6)
High-stressed at T2 (only B region), *n* (%)		113 (13.4)	33 (14.7)	26 (11.9)	47 (9.2)	18 (19.6)	26 (17.3)	20 (15.3)	283 (13.1)

T1, Survey in October 2018; T2, Survey in October 2019.

Table 2 shows the results of univariate and multivariate logistic regression for both male and female when identifying high stress at T2 based on criteria A and B. The percentages of female and male who were identified as highly stressed at T2 and who worked 41–50 h per week at both T1 and T2 (reference) were 11.2 and 13.6 %, respectively. In Table 3, crude ORs for high stress among female were higher for those who worked 41–50 h per week at T1 and more than 50 h at T2, for those who worked more than 50 h per week at T1 and 35–40 h/41–50 h per week at T2, and for those who worked more than 50 h per week at both T1 and T2 [OR = 2.20 [95% CI, 1.25–3.88]: OR = 2.07 [95% CI, 1.28–3.34]: OR = 1.78 [95% CI, 1.06–3.00], respectively]. However, among male, none of the categories of working hours showed statistically significant results.

**Table 2:** Summary of backgrounds and characteristics of male workers.

**Working hours** **(Hours per week)**	**T1 (October 2018)**	**35~40**		**41~50**			**51~**		**Total (male)**
	**T2 (October 2019)**	**35**~**40**	**41**~**50/51**~	**35**~**40**	**41**~**50**	**51**~	**35**~**40/41**~**50**	**51**~	
n		344	153	152	493	133	165	267	1,707
Age, mean (standard deviation)		48.7 (10.7)	44.7 (10.3)	45.6 (10.1)	43.8 (10.5)	43.4 (9.5)	43.9 (10.0)	43.3 (10.2)	44.9 (10.5)
Education year ≥16 years, *n* (%)		229 (66.6)	106 (69.3)	110 (72.4)	362 (73.4)	97 (72.9)	127 (77.0)	206 (77.2)	1,237 (72.5)
Professional, *n* (%)		158 (45.9)	85 (55.6)	84 (55.3)	299 (60.7)	86 (64.7)	102 (61.8)	183 (68.5)	997 (58.4)
**Years of experience**, ***n*** **(%)**									
≤ 5 years		126 (36.6)	64 (41.8)	53 (34.9)	175 (35.5)	48 (36.1)	56 (33.9)	84 (31.5)	606 (35.5)
6~10 years		59 (17.2)	30 (19.6)	25 (16.5)	94 (19.1)	25 (18.8)	34 (20.6)	53 (19.9)	320 (18.8)
11~20 years		72 (20.9)	25 (16.3)	37 (24.3)	112 (22.7)	33 (24.8)	40 (24.2)	60 (22.5)	379 (22.2)
≥21 years		87 (25.3)	34 (22.2)	37 (24.3)	112 (22.7)	27 (20.3)	35 (21.2)	70 (26.2)	402 (23.6)
**Income change**, ***n*** **(%)**									
No change		183 (53.2)	69 (45.1)	74 (48.7)	271 (55.0)	62 (46.6)	90 (54.6)	137 (51.3)	886 (51.9)
Increased		65 (18.9)	34 (22.2)	34 (22.4)	102 (20.7)	40 (30.1)	34 (20.6)	60 (22.5)	369 (21.6)
Decreased		44 (12.8)	26 (17.0)	21 (13.8)	63 (12.8)	17 (12.8)	15 (9.1)	40 (15.0)	226 (13.2)
No answer		52 (15.1)	24 (15.7)	23 (15.1)	57 (11.6)	14 (10.5)	26 (15.8)	30 (11.2)	226 (13.2)
The number of roommates changed, *n* (%)		19 (5.5)	11 (7.2)	8 (5.3)	25 (5.1)	8 (6.0)	14 (8.5)	15 (5.6)	100 (5.9)
With treatment, *n* (%)		94 (27.3)	30 (19.6)	33 (21.7)	88 (17.9)	22 (16.5)	25 (15.2)	44 (16.5)	336 (19.7)
High-stressed at T2 (all the region), *n* (%)		43 (12.5)	24 (15.7)	22 (14.5)	67 (13.6)	22 (16.5)	20 (12.1)	46 (17.2)	244 (14.3)
High-stressed at T2 (only B region), *n* (%)		42 (12.2)	22 (14.4)	20 (13.2)	59 (12.0)	16 (12.0)	19 (11.5)	38 (14.2)	216 (12.7)

T1, Survey in October 2018; T2, Survey in October 2019.

**Table 3:** Estimated odds ratios for high stress based on the Brief Job Stress Questionnaire by gender.

		**Female**						**Male**					
		**Crude**			**Adjusted**			**Crude**			**Adjusted**		
		**OR**	**95%CI**	***p*** **value**	**OR**	**95% CI**	***p*** **value**	**OR**	**95% CI**	***p*** **value**	**OR**	**95% CI**	***p*** **value**
Working hours (hours per week)	35~40 at T1 and 35~40 at T2	1.33	0.95–1.86	0.096	1.31	0.93–1.84	0.119	0.91	0.60–1.37	0.646	0.84	0.55–1.27	0.413
	35~40 at T1 and 41~50/51~ at T2	1.51	0.96–2.37	0.073	1.45	0.92–2.29	0.108	1.18	0.71–1.96	0.515	1.14	0.68–1.90	0.616
	41~50 at T1 and 35~40 at T2	1.17	0.72–1.89	0.527	1.13	0.70–1.84	0.613	1.08	0.64–1.81	0.783	1.04	0.62–1.76	0.871
	41~50 at T1 and 41~50 at T2	1.00	reference		1.00	reference		1.00	reference		1.00	reference	
	41~50 at T1 and 51~ at T2	2.20	1.25–3.88	0.006	2.09	1.18–3.70	0.012	1.26	0.75–2.13	0.388	1.29	0.76–2.18	0.352
	51~ at T1 and 35~40/41~50 at T2	2.07	1.28–3.34	0.003	1.86	1.14–3.03	0.013	0.88	0.51–1.50	0.630	0.86	0.50–1.47	0.570
	51~ at T1 and 51~ at T2	1.78	1.06–3.00	0.030	1.69	1.00–2.87	0.051	1.32	0.88–1.99	0.179	1.37	0.91–2.07	0.134
Years of experience	≤ 5 years				1.00	reference					1.00	reference	
	6~10 years				1.08	0.79–1.48	0.613				0.74	0.50–1.17	0.147
	11~20 years				0.67	0.49–0.93	0.017				0.99	0.68–1.43	0.936
	≥ 21 years				0.68	0.46–1.00	0.048				1.06	0.73–1.54	0.768
Education years	< 16 years				1.00	reference					1.00	reference	
	≥ 16 years				1.12	0.87–1.43	0.388				0.77	0.57–1.04	0.091
Treatment	Without medication				1.00	reference					1.00	reference	
	With medication				0.87	0.53–1.43	0.580				1.07	0.76–1.51	0.709
Income change	No change				1.00	reference					1.00	reference	
	Increased				1.08	0.77–1.51	0.663				0.88	0.62–1.26	0.479
	Decreased				1.54	1.09–2.17	0.015				0.92	0.60–1.40	0.698
	No answer				1.32	0.96–1.81	0.089				1.00	0.66–1.52	0.988
Someone who lives with you	No change				1.00	reference					1.00	reference	
	Increased/decreased				1.42	0.92–2.20	0.118				2.07	1.28–3.35	0.003
Type of work	General clerk				1.00	reference					1.00	reference	
	Professional				1.14	0.87–1.50	0.347				0.76	0.57–1.02	0.066

OR, odds ratio; 95% CI, 95% confidence interval; T1, Survey in October 2018; T2, Survey in October 2019.

When analyzing other variables together with working hours, ORs for high stress among female were higher for those who worked 41–50 h per week at T1 and more than 50 h at T2, and for those who worked more than 50 h per week at T1 and 35–40 h/41–50 h per week at T2 (OR = 2.09 [95% CI, 1.18–3.70]: OR = 1.86 [95% CI, 1.14–3.03], respectively). In addition, ORs for high stress among female were higher for those whose income had decreased at T2 (OR = 1.54 [95% CI, 1.09–2.17]). ORs for high stress among female were lower for those whose years of experience were more than 10 years and more than 20 years [OR = 0.67 [95% CI, 0.49–0.93]: OR = 0.68 [95% CI, 0.46–1.00], respectively]. On the other hand, among male, none of the categories of working hours showed statistically significant results, except that ORs for high stress were significantly higher among male whose number of roommates had increased or decreased (OR = 2.07 [95% CI, 1.28–3.35]).

The direction of ORs did not change in the sensitivity analyses in which the cut-off for working hours was changed (Supplementary Table S1).

Table 4 shows results of univariate and multivariate logistic regression of both male and female participants when identifying high stress at T2 based only on criterion A. The rate of female and male participants who were identified as highly stressed at T2 and who worked 41–50 h per week both at T1 and T2 (reference) were 9.2 and 12.0%, respectively. In addition to categories of working hours which showed statistically significant data in Table 4, crude ORs for high stress among female were higher for those who worked 35–40 h per week at T1 and T2, and for those who worked 35–40 h per week at T1 and 41–50/51 h per week at T2 as well [OR = 1.52 [95% CI, 1.06–2.18]; OR = 1.69 [95% CI, 1.05–1.72], respectively]. When analyzing other variables, ORs for high stress among male were lower for those whose type of work was professional.

**Table 4:** Estimated odds ratios for high stress based on (B) psychological and physical stress responses category of the Brief Job Stress Questionnaire by gender.

		**Female**						**Male**					
		**Crude**			**Adjusted**			**Crude**			**Adjusted**		
**T1**	**T2**	**OR**	**95%CI**	***p*** **value**	**OR**	**95% CI**	***p*** **value**	**OR**	**95% CI**	***p*** **value**	**OR**	**95% CI**	***p*** **value**
35–40	35–40	1.52	1.06–2.18	0.022	1.50	1.04–2.15	0.030	1.02	0.67–1.56	0.916	0.95	0.62–1.47	0.830
	41~50/51~	1.69	1.05–1.72	0.031	1.64	1.02–2.65	0.043	1.24	0.73–2.09	0.432	1.19	0.70–2.03	0.526
41~50	35~40	1.33	0.80–2.21	0.270	1.31	0.78–2.18	0.304	1.11	0.65–1.92	0.696	1.08	0.62–1.86	0.794
	41~50	1.00	reference		1.00	reference		1.00	reference		1.00	reference	
	51~	2.39	1.32–4.34	0.004	2.26	1.24–4.13	0.008	1.01	0.56–1.81	0.984	1.01	0.56–1.83	0.967
51~	35~40/41~50	2.06	1.23–3.46	0.006	1.86	1.10–3.15	0.020	0.96	0.55–1.66	0.876	0.92	0.53–1.61	0.772
	51~	1.77	1.01–3.11	0.047	1.68	0.95–2.97	0.075	1.22	0.79–1.89	0.372	1.26	0.81–1.96	0.310
Years of experience	≤ 5 years				1.00	reference					1.00	reference	
	6~10 years				1.12	0.81–1.54	0.510				0.74	0.47–1.15	0.181
	11~20 years				0.65	0.46–0.92	0.014				1.06	0.72–1.56	0.778
	≥21 years				0.74	0.50–1.10	0.142				1.16	0.78–1.72	0.472
Education years	< 16 years				1.00	reference					1.00	reference	
	≥16 years				1.18	0.91–1.54	0.201				0.80	0.58–1.09	0.156
Under treatment	Without treatment				1.00	reference					1.00	reference	
	With treatment				0.88	0.52–1.47	0.621				0.90	0.62–1.31	0.582
Income change	No change				1.00	reference					1.00	reference	
	Increased				1.14	0.80–1.63	0.454				0.97	0.67–1.42	0.893
	Decreased				1.57	1.10–2.26	0.014				0.91	0.58–1.43	0.670
	No answer				1.29	0.93–1.81	0.131				1.13	0.73–1.74	0.578
Roommate	No change				1.00	reference					1.00	reference	
	Increased/decreased				1.41	0.89–2.23	0.142				2.12	1.29–3.48	0.003
Type of work	General clerk				1.00	reference					1.00	reference	
	Professional				1.08	0.81–1.44	0.608				0.74	0.54–1.00	0.050

OR, odds ratio; 95% CI, 95% confidence interval; T1, Survey in October 2018; T2, Survey in October 2019.

## 4. Discussion

In the present study, we used a nationally representative sample of white-collar workers in Japan to examine the prevalence of those who had low stress at the first time point but had high stress 1 year later. We considered the relationship between changes in working hours and stress levels. The incidence of high stress for male and female in this population based on criteria A and B was 14.6 and 14.3%, respectively, reflecting no gender difference in the number of new cases of stress per year. Our results were consistent with the prevalence of stress studied cross-sectionally (20). We evaluated an association between changing working hours and high stress in a longitudinal design, which had previously been proposed only in a cross-sectional manner (21). Although, as expected, high stress occurred when working hours increased in a longitudinal design, more than 11.5% of those whose working hours either decreased or remained the same were highly stressed 1 year later. Because stress levels can change over time, it is important to monitor these levels in workers (12, 13) to prevent accidents, injuries, and worsening health (5).

Regarding the degree of increasing stress between categories of changes in working hours, an interesting result was that in female, there was a greater increase in stress compared to the reference group in several working categories; contrastingly, none of the working hour categories showed statistically significant data among male. One potential reason for this phenomenon could be that, as the previous study mentioned, long working hours were related to the risk of high stress levels; women were also more likely to respond negatively to high job strain (22) than were men. Higher job strain due to increased working hours can predispose women to higher stress. Additionally, the effect of increased work intensity on worker satisfaction can be buffered by high autonomy (23, 24). In this study, male made up a higher proportion of professional workers than did female, which is common. Professional workers may have higher autonomy (23). Being a professional worker is a buffering factor between long working hours and high stress, given that ORs for high stress among male were lower among those who were professional workers when high stress was evaluated based only on criterion A. Similar results were observed in the previous study: among IT company workers aged 40 years or younger, there were no significant differences in mental and physical status between overtime work (>45 h/month overwork) and non-overtime work groups in both sexes (25). IT professionals often find themselves coerced into accepting high workloads under high pressure to shorten the time to market, as well as to learn new skills because of rapid advances in technology (26, 27). However, elevated job strain can be lessened by providing job autonomy and skill variation to employees (28). Considering professionals have higher job autonomy than do other occupations, high job autonomy could mitigate the negative effects of increased working hours on stress levels.

Among female, both increased and decreased working hours caused a greater increase in stress than when working hours were stable. Women's working hours could have been reduced involuntarily, resulting in high stress. Furthermore, employers might have cut their working hours due to poor job performance related to distress associated with work–family conflict (29, 30). Indeed the interaction between conditions at work and at home plays an important role in determining the health of women employees, whereas the health of employed men is determined more selectively by working conditions (31). This difference is expected to be more evident in Japan, where women are still responsible for performing domestic chores according to traditional gender roles (32). In fact, among middle-aged Japanese working women, regular employees have poorer self-reported health than non-regular employees, a difference attributed to experiencing more strain related to work–family conflict (33). Additionally, in various countries, women face an increased risk of health problems due to long working hours and greater family responsibilities (34, 35). Hence, work–family conflict could induce high levels of stress in women employees. To reduce such conflict, employers could willingly adopt flexible work schedules, which could promote a better balance between work and personal life (36).

Incidence of high stress might directly and indirectly be related to economic status. In women, reduction of income could be related to increasing stress, and lead to dissatisfaction at work; this situation can adversely affect employees' mental and physical health and their overall wellbeing (37). This is also true in men; ORs for high stress among men were significantly higher for those whose number of roommates increased or decreased. Studies show that being separated is associated with a high prevalence of psychological distress in both men and women (38). One reason high stress was also observed in male whose roommates had increased could be related to male gender roles at home. In Japan, gender roles are still characterized by the model of a strong man being the breadwinner and by women's low level of participation in the workforce (39). Marriage has been shown to enhance negative effects of unemployment on men's mental health but act as a buffer among women; thus, it is reasonable to suppose that marriage can stimulate men's sense of responsibility, which could predispose them to high stress (40).

When evaluating the high-stress state based only on criterion A, the incidence in female and male was still 13.1 and 12.7%, respectively. A previous study also showed a subtle difference in prevalence between criteria A and B and using only criterion A. In a financial company comprising 7,356 male and 7,362 female employees, the prevalence of high stress based on criteria A and B of the BJSQ was 5.6% for male and 15.0% for female. When evaluated based on criterion A, the prevalence was 4.5% for male and 13.2% for female (19). Combined with the results of previous studies, our results suggest that over the course of 1 year, Japanese employees can progress to high stress levels severe enough to cause physical reactions. Studies have indicated that workers in Japan working more than 50 h per week have an increased risk of occupational accidents (41), and workers in the EU who work more than 55 h per week have an increased risk of stroke, atrial fibrillation, and several other diseases (42–44). Notably, in this study, the increase in stress occurred in all categories for male, although there was no difference among the categories of the changes in working hours. The effect on objective outcome may be different when stress increases in situations with increased working hours and in other situations. Further studies are needed to evaluate long-term outcomes in Japan.

In this study, we investigated working hours to identify groups prone to high stress among homogeneous groups not in a high stress state, especially regarding changes in working hours. In recent years, efforts have been made to reduce the working hours in Japan (16); however, the amount of reduction varies by industry, and in some occupations, the workload increased due to the COVID-19 pandemic (45). From a preventive perspective, it might be important to not only manage working hours according to job demands, but also create a positive workplace by, for example, providing co-worker support and job autonomy, and allowing flexible work styles. It is also crucial to consider how to manage high-stress situations (46). For such a study, it is also important to evaluate the continuous state of high stress and changes in the previous stress state based on a homogeneous group.

Although this study provides new insights into the incidence of high stress over 1 year, and how changes in stress depend on changes in working hours by gender, several limitations should be mentioned. First, recall bias may have occurred because working hours per week were self-reported; those identified as highly stressed in the second survey were more likely to report longer working hours than they actually performed. Second, there is the possibility of selection bias; responses to stress in a population that can respond to two web questionnaires may contain bias. However, because the sampling was based on the distribution of the population by age, gender, and industry, the results were consistent with high stress rates in other studies. Third, this study evaluated average working hours during the last 6 months, which made it impossible to follow in more detail how working hours changed. Fourth, our results regarding the effect of changes in working hours on stress levels may not be applicable to the current working environment where many employees have been obliged to work from home, changed their commute time, and transportation methods due to the COVID-19 pandemic. However, working from home has a negative effect on employees' physical and mental health (47). It is important to identify other variables, besides working hours, that can contribute to high stress. Lastly, we did not show a causal relationship between changes in working hours and stress, because we did not perform any interventions to increase or decrease working hours. Thus, how stress changes after intentional increases in working hours remains to be seen.

## Data availability statement

The datasets presented in this article are not readily available because they contain information that could compromise the privacy of research participants, based on the “Ethical Guidelines for Medical and Health Research involving Human Subjects” by the Japanese government. Requests to access the datasets should be directed to corresponding author.

## Ethics statement

The studies involving human participants were reviewed and approved by the Institutional Review Board of the Jikei University School of Medicine, Tokyo, Japan, in 2018 [No. 30-153(9174)]. The patients/participants provided their written informed consent to participate in this study.

## Author contributions

TY and KT: conceptualization. TY: data collection. TA and KT: methodology. MO and TA: statistical analyses and writing–original draft. KT: supervising. All authors critically revised the manuscript, contributed to the interpretation of the data, and approved the final manuscript.
