# Supplementary material for: Do changes in working hours increase stress in Japanese white-collar workers?
Source: Front Public Health. 2023 Feb 1;11:1076024. doi: 10.3389/fpubh.2023.1076024 (PMC9928859; doi:10.3389/fpubh.2023.1076024)
Supplement: Supplementary file 1 [file Table_1.DOCX]

Supplementary Material

Do changes in working hours increase stress in Japanese white-collar workers?

Masaki Ozawa, Tatsuhiko Anzai, Takashi Yamauchi, Kunihiko Takahashi*

*** Correspondence:** Kunihiko Takahashi: kunihikot.dsc@tmd.ac.jp

Table S1. Sensitivity analysis: Estimated odds ratios for high stress based on the Brief Job Stress Questionnaire by gender modifying the working hours

|  |  | **Female** |  |  |  | **Male** |  |  |
| --- | --- | --- | --- | --- | --- | --- | --- | --- |
|  |  | Adjusted* | | |  | Adjusted* | | |
|  |  | OR | 95% CI | p value |  | OR | 95% CI | p value |
| Working hours  (hours per week) | 35~40 at T1 and 35~40 at T2 | 1.11 | 0.82 to 1.84 | 0.477 |  | 0.82 | 0.56 to 1.21 | 0.313 |
|  | 35~40 at T1 and 41~60/61~ at T2 | 1.23 | 0.81 to 2.29 | 0.334 |  | 1.11 | 0.68 to 1.80 | 0.674 |
|  | 41~60 at T1 and 35~40 at T2 | 0.98 | 0.64 to 1.51 | 0.940 |  | 0.95 | 0.59 to 1.54 | 0.847 |
|  | 41~60 at T1 and 41~60 at T2 | 1.00 | reference |  |  | 1.00 | reference |  |
|  | 41~60 at T1 and 61~ at T2 | 2.86 | 1.46 to 5.59 | 0.002 |  | 1.22 | 0.62 to 2.42 | 0.558 |
|  | 61~ at T1 and 35~40/41~60 at T2 | 1.83 | 0.97 to 3.43 | 0.061 |  | 1.34 | 0.77 to 2.37 | 0.300 |
|  | 61~ at T1 and 61~ at T2 | 1.09 | 0.37 to 3.27 | 0.873 |  | 1.57 | 0.85 to 2.88 | 0.145 |

OR, odds ratio; 95%CI, 95% confidence interval; T1, Survey in October 2018; T2, Survey in October 2019

Adjusted with years of experience, education years, treatment, income change, roommate, and type of work.
